# The evolving epidemiology of rotavirus A infection in Brazil a decade after the introduction of universal vaccination with Rotarix®

**DOI:** 10.1186/s12887-019-1415-9

**Published:** 2019-01-31

**Authors:** Filipe A. Carvalho-Costa, Rosane M. S. de Assis, Alexandre M. Fialho, Irene T. Araújo, Marcelle F. Silva, Mariela M. Gómez, Juliana S. Andrade, Tatiana L. Rose, Tulio M. Fumian, Eduardo M. Volotão, Marize P. Miagostovich, José Paulo G. Leite

**Affiliations:** 1 Laboratory of Comparative and Environmental Virology, Oswaldo Cruz Institute, Oswaldo Cruz Foundation, Av. Brasil 4365 Pavilhão Hélio e Peggy Pereira, Manguinhos, Rio de Janeiro, Rio de Janeiro Brazil; 2 Laboratory of Epidemiology and Molecular Systematics, Oswaldo Cruz Institute, Oswaldo Cruz Foundation, Av. Brasil 4365 Pavilhão Leonidas Deane, Manguinhos, Rio de Janeiro, Rio de Janeiro Brazil; 3 Regional Office Fiocruz Piauí. Rua Magalhães Filho, n° 519, Centro/Norte, Teresina, Piauí Brazil

**Keywords:** Rotavirus, Monovalent vaccine, Genotypes, Epidemiology

## Abstract

**Background:**

Brazil introduced the monovalent rotavirus vaccine (Rotarix**®**) in 2006. This study aimed to assess the epidemiology and genotype distribution of species-A rotavirus (RVA) in Brazil, comparing the pre- and post-vaccination periods.

**Methods:**

Laboratory-based RVA surveillance included 866 municipalities in 22 Brazilian states, over a 21-year period. A total of 16,185 children with diarrheal diseases (DD) aged up to 12 years between 1996 and 2005 (pre-vaccination period, *n* = 7030) and from 2006 to 2017 (post-vaccination period, *n* = 9155) were enrolled. RVA was detected using ELISA immune assay and/or polyacrylamide gel electrophoresis and genotyped using nested PCR and/or nucleotide sequencing. RVA-positivity and genotypes detection rates were compared in distinct periods and age groups and Rotarix vaccination status.

**Results:**

RVA-positivity in pre- and post-vaccination periods was, respectively: 4–11 months bracket, 33.3% (668/2006) and 16.3% (415/2547) (*p* <  0.001); 12–24 months, 28.2% (607/2154) and 22.2% (680/3068) (*p* <  0.001); 25–48 months, 17.4% (215/1235) and 29.4% (505/1720) (*p* <  0.001). Genotypes distribution in the pre- and post-vaccination periods was, respectively: G1P [8]/G1P[Not Typed], 417/855 (48.8%) and 118/1835 (6.4%) (*p* <  0.001); G2P [4]/G2P[NT], 47/855 (5.5%) and 838/1835 (45.7%) (*p* <  0.001); G3P [8]/G3P[NT], 55/855 (6.4%) and 253/1835 (13.8%) (*p* <  0.001); G9P [8]/G9P[NT], 238/855 (27.8%) and 152/1835 (8.3%) (*p* <  0.001); G12P [8]/G129P[NT], 0/871 (0%) and 249/1835(13.6%) (*p* <  0.001). Concerning infants aged 4–11 months, RVA frequency in fully vaccinated and non-vaccinated individuals was 11.9% (125/1052) and 24.5% (58/237) (*p* <  0.001), respectively. In children aged 12–24 months, RVA detection rate was 18.1% (253/1395) and 29.6% (77/260) (*p* <  0.001), for the vaccinated and non-vaccinated individuals, respectively (*p* <  0.001).

**Conclusions:**

RVA infection was significantly less frequent in children aged ≤2 years with DD after implementing vaccination, mainly among vaccinated children. It was also observed a decrease of P [8] circulation and emergence of G2P[4] in 2005, and afterwards in the post-vaccine era, with spreading of G12P[8] in 2014–2015 and of G3P[8] in 2017. Continuous RVA surveillance must be carried out in this scenario.

## Background

Diarrheal diseases (DD) are one of the leading causes of death in children ≤5 years old, accounting for almost 10% of deaths in this age group [1–4]. Globally, rotavirus A (RVA), norovirus (NoV) genogroup II, astroviruses (HAstV), *Campylobacter* sp., *Cryptosporidium* sp*.*, enterotoxigenic *Escherichia coli*, and *Shigella sp.* are the most prevalent agents of DD [5–9]. The Global Rotavirus Surveillance Network has shown that, although approximately 40.3% of DD cases can be attributed to RVA globally; in countries of the Americas with universal vaccination this proportion is 12.2% [10]. In China, a country where the two licensed RVA vaccines were not included in the routine vaccination schedule, the overall rate of RVA positivity in children with DD is 30% [11].

RVA possesses an RNA genome with 11 gene segments and is commonly classified using a binary system based on two outer and most immunogenic capsid proteins [12]. Among the most prevalent RVA genotypes, G1P[8], G3P[8], G4P[8], G9P[8] and G12P[8] belong to the Wa-like genomic constellation, while G2P[4] belongs to the DS-1-like constellation [13]. These two major genomic assemblages display nucleotide sequence identities varying from 75 to 90% [13, 14].

In 2017, Brazil completed a decade of vaccine implementation (the attenuated monovalent G1P[8] vaccine (Rotarix®, RV1) in the National Immunization Program (NIP), which has expanded substantially in the last years. Vaccination with RV1 consists of two doses. Infants aged 6 weeks to 8 months are vaccinated. The first dose should be given until the age of 3 months and 15 days, and the last dose up to 7 months and 29 days.

According to the World Health Organization and the Pan American Health Organization (WHO/PAHO), RVA vaccines are strategic to reduce DD burden, along with oral rehydration, breastfeeding, zinc administration and improvement of sanitation [15]. So far, more than 81 countries introduced RVA vaccination since October 2016. From these, 63 countries introduced RV1 and 18 countries implemented vaccination with the pentavalent vaccine RotaTeq® (RV5) (four countries introduced both RV1 and RV5). Currently, the Global Alliance for Vaccines and Immunization (GAVI), supports RVA vaccination in 45 developing countries (https://www.gavi.org/results/countries-approved-for-support/).

A meta-analysis of RVA surveillance studies – including data from countries that participated in the WHO RVA surveillance network from 2008 to 2013 – estimated a reduction on a global scale from 528,000 to 215,000 RVA-associated deaths in children ≤5 years old from 2000 to 2013. In the same period, the RVA detection rates in children with DD declined from 42.5 to 37.3% [16]. The positive impact of RVA vaccination on DD-associated hospitalizations and deaths has been well demonstrated in Brazil and several other Latin American countries [17, 18]. In Brazil, effectiveness is higher among infants aged up to 12 months, decreasing in older children [19–21].

In this study, we accessed the impact of Rotarix after ten years of its implementation in the NIP in Brazil. For this propose, we explored RVA detection rates and genotype distribution in DD samples collected from children in the pre- and post-vaccination periods.

## Methods

### Study design and laboratory-based rotavirus A surveillance

This is a retrospective study with surveillance data. The RVA laboratory-based surveillance is a public health surveillance system which aims to monitor the circulation of different RVA genotypes and lineages in distinct Brazilian regions. Fecal samples collected from patients with DD are sent together with clinical-epidemiological records to the Regional Rotavirus Reference Laboratory - Laboratory of Comparative and Environmental Virology (RRRL-LVCA) which is one of the laboratories of this system and received fecal samples from states during the study period. Samples received by RRRL-LVCA are also tested for other enteric viruses, including NoV, HAstV and adenoviruses, as well as emerging viruses such as human bocaviruses and aichivirus. The analyzed variables were: age of the child, month, season and year in which the case of DD occurred, vaccination status in relation to RV1, positivity for RVA infection and the result of RVA genotyping. Although there are no individual data on DD cases, clinically detailing the episodes, it can be stated that they were all severe enough to motivate attending the primary health care system or a hospital. Frequency of RVA infection (detection rate) was calculated as the number of RVA-positive subjects / the number of DD cases analyzed X 100, in distinct age groups and vaccination periods, as well as among vaccinated and non-vaccinated children. Fecal samples were obtained from children aged up to 12 years old with DD (*n* = 16,185) in 866 municipalities from 22 out of 27 Brazilian states, covering a 21 years period, from 1996 to 2005 (pre-vaccination period) and from 2006 to 2017 (post-vaccination period), as presented in the Table 1. States that have not submitted samples have other laboratories as a reference for RVA diagnosis and genotyping. Samples and forms with demographic and epidemiologic information, as well as RV1 vaccination status were sent to the RRRL-LVCA, Oswaldo Cruz Institute, Fiocruz, Ministry of Health. A total of 8179 samples were sent by a network of State Public Health Laboratories which received fecal samples collected in health units within the Unified Health System. The remaining 8006 samples were sent directly to the RRRL-LVCA by pediatric hospitals, day care centers, and primary care health units. The DD cases included in this study do not discriminate between hospitalized and outpatient children. However, all the children needed to be hydrated orally or intravenously in their units of origin and presented sufficient severity leading to clinic or hospital visit.

**Table 1:** Number of fecal samples analyzed through laboratory-based rotavirus surveillance by state, in the pre- and pos-vaccination periods, Brazil, 1996–2017

	No. of fecal samples analyzed from 1996 to 2005 (pre-vaccination period)	No. of fecal samples analyzed from 2006 to 2017 (post-vaccination period)
Region Southeast		
Espírito Santo	99	393
Minas Gerais	19	505
Rio de Janeiro	3811	1786
Region Northeast		
Alagoas	–	121
Bahia	2224	653
Ceará	–	205
Maranhão	–	863
Paraíba	–	23
Pernambuco	23	464
Rio Grande do Norte	–	28
Sergipe	–	783
Region South		
Paraná	82	–
Rio Grande do Sul	387	2326
Santa Catarina	4	947
Region North		
Acre	283	25
Amazonas	–	6
Pará	–	18
Rondônia	–	4
Region Central-West		
Federal District	33	2
Goiás	40	–
Mato Grosso do Sul	22	2
Mato Grosso	3	1

### Rotaviruses A detection and genotyping

Enzyme immunoassay kits (EIARA®, Biomanguinhos, Rio de Janeiro, Rio de Janeiro, Brazil; Premier Rotaclone®, Meridian Bioscience Inc., Cincinatti, Ohio, USA or Ridascreen Rotavirus®, R-Biopharm, Darmstadt, Hesse, Germany) and polyacrylamide gel electrophoresis (PAGE) were used for RVA detection in 10% fecal suspensions in phosphate-buffered saline pH 7.4 [22]. Nucleic acids were extracted from clarified stool supernatants using the silica-based method previously described by Boom et al. (1990) or the QIAamp Viral RNA Mini Kit® (QIAGEN, Valencia, CA, USA) according to the manufacturer’s instructions. Positive samples were G- and P-genotyped by semi-nested multiplex reverse transcription-polymerase chain reaction (RT-PCR) and/or by genome sequencing. The RVA dsRNA was reverse transcribed and amplified with a pair of consensus primers directed to a conserved region within the genes codifying VP4 and VP7 proteins. The amplicon fragments of 876 bp and 904 bp for VP4 and VP7, respectively, were used as a template in a second PCR amplification with a pool of genotype-specific primers. Milli-Q water and reference RVA-positive fecal sample were used as negative and positive controls, respectively, and recommended manipulations of PCR procedures were carried out to avoid false-positive results.

### Statistical analysis

Statistical analyses were performed using SPSS® (IBM Corp., Armonk, NY, USA). Frequencies of RVA detection in different age groups, as well as rates of detection of distinct genotypes in the pre- and post-vaccination periods were compared using the Chi-square test. We calculated odds ratios (ORs) of RV1 vaccination among RVA-positive and RVA-negative children in distinct age groups and used Fisher’s exact test to verify the statistical significance of the associations. In all analyses a *p*-value inferior to 0.05 was considered statistically significant.

## Results

### Rotaviruses A detection rates in children with diarrheal disease

**Fig. 1:**
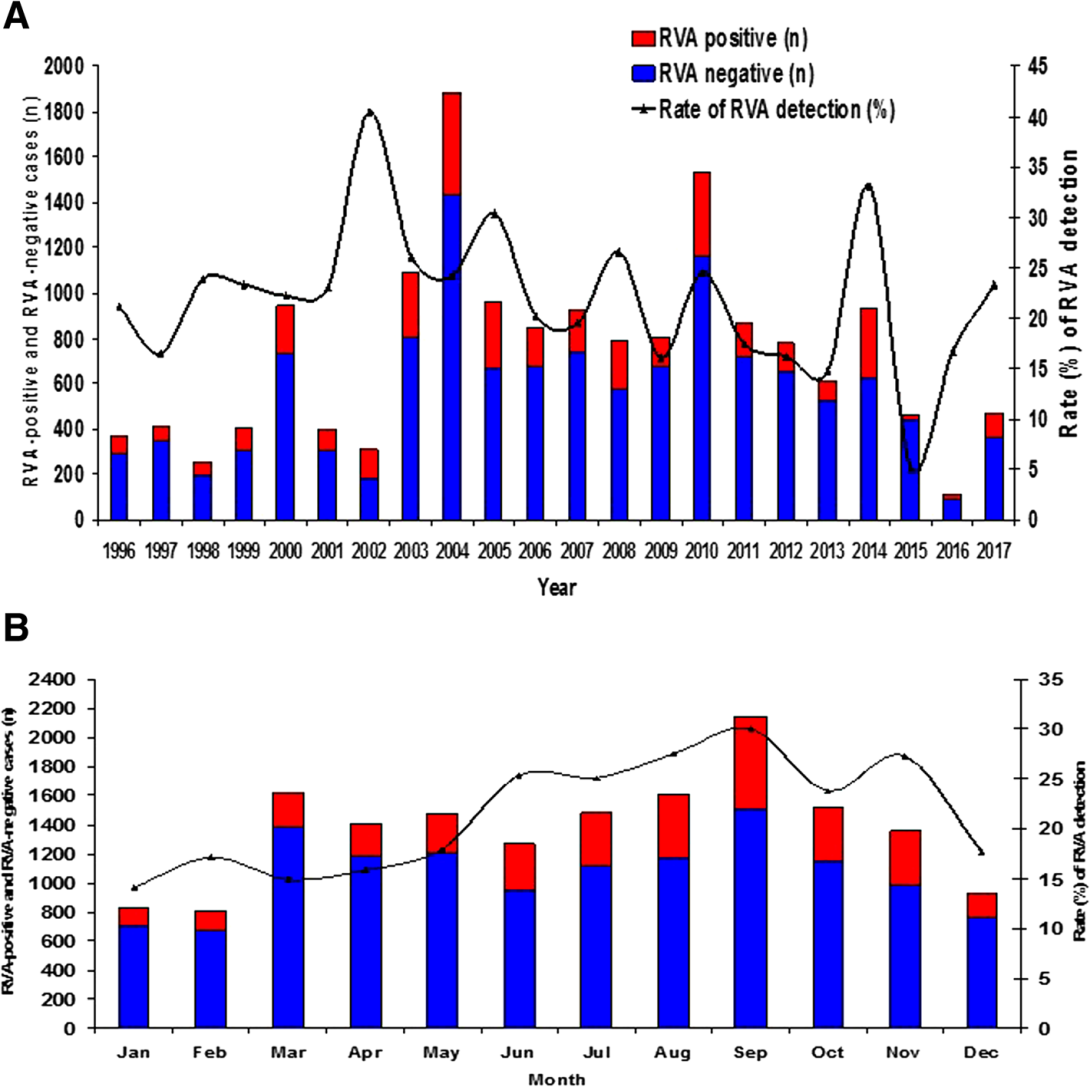
**a** and **b** Laboratory-based rotaviruses A (RVA) surveillance in Brazil, 1996–2017. Rate of RVA detection by year (1A) and month (1B), and number of RVA-positive and RVA-negative samples, collected in 22 out of 27 Brazilian states

**Fig. 2:**
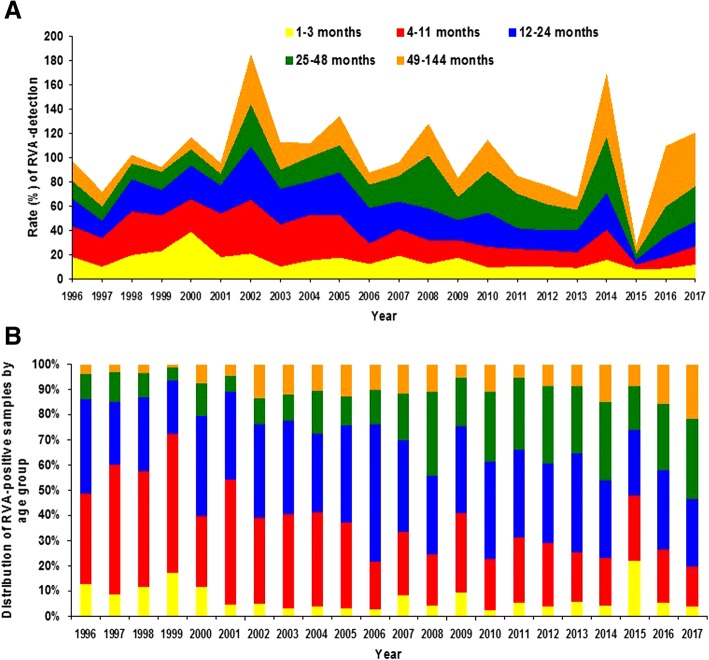
**a** and **b** Laboratory-based rotaviruses A (RVA) surveillance in Brazil, 1996–2016, in 22 out of 27 Brazilian states. Rate of RVA detection by year in distinct age groups (2A) and distribution of RVA-positive samples by age group (2B)

RVA-positivity by year and month over the 21-year period is depicted in Fig. 1a Frequency of RVA infection by year in distinct age groups and distribution of RVA infections by age groups in distinct years are presented in Fig. 2a and b, respectively.

As presented in Table 2, RVA detection rates decreased significantly in children aged up to 24 months, e.g. among infants aged 1–3 months, RVA positivity decreased from 18.4% (108/587) to 12.5% (91/729) (*p* = 0.003). RVA detection rate in children was reduced in the 4–11 months bracket from 33.3% (668/2006) in the pre-vaccination period to 16.3% (415/2547) in the post-vaccination period (*p* <  0.001). In addition, frequency of infection with RVA reduced from 28.2% (607/2154) to 22.2% (680/3068) (p <  0.001) among children aged 12–24 months, after implementing universal vaccination with RV1. An increase in RVA detection rates was observed among older children with DD following the introduction of RV1. In the group of children aged 25–48 months, RVA was detected in 17.4% (215/1235) and in 28.3% (505/1782) comparing the pre and post-vaccination periods (*p* <  0.001). Among children aged 49–144 months, RVA detection increased from 15.6% (164/1048) to 21.3% (332/1556) (*p* <  0.001) (data not shown). Despite the increase in the absolute number of RVA-positive and total DD cases in the post-vaccination period (reflecting the intensification of surveillance in this period), the overall detection rate of RVA infection among children aged 1–144 months with DD decreased from 25.1% (1762/7030) before vaccine introduction to 20.8% (1903/9155) in the period from 2006 to 2017 (p <  0.001).

**Table 2:** Rate of rotavirus A (RVA) detection by age group and rate of detection of distinct RVA genotypes in the ore- and post-vaccination periods in Brazil, 1996–2017

	1996 to 2005 (pre-vaccination period)	2006 to 2017 (post-vaccination period)	*p*-value (chi-square test)
Age group			
1–3 months	108/587 (18.4%)	91/729 (12.5%)	0.003
4–11 months	668/2006 (33.3%)	415/2547 (16.3%)	< 0.001
12–24 months	607/2154 (28.2%)	680/3068 (22.2%)	< 0.001
25–48 months	215/1235 (17.4%)	505/1782 (28.3%)	< 0.001
49–144 months	164/1048 (15.6%)	332/1556 (21.3%)	< 0.001
Total	1762/7030 (25.1%)	1903/9155 (20.8%)	< 0.001
RVA^a^ genotypes			
G1P [8] / G1P[NT^b^]	417/855 (48.8%)	118/1835 (6.4%)	< 0.001
G2P [4] / G2P[NT]	47/855 (5.5%)	838/1835 (45.7%)	< 0.001
G3P [8] / G3P[NT]	55/855 (6.4%)	253/1835 (13.8%)	< 0.001
G9P [8] / G9P[NT]	238/855 (27.8%)	152/1835 (8.3%)	< 0.001
G12P [8] / G12P[NT]	–	249/1835 (13.6%)	< 0.001

^a^Rotavirus A; ^b^Not typed

By analyzing RVA detection throughout the seasons, we observed lower detection rates during the summer (16.2% [422/2601]) and autumn (16.6% [724/4362]) compared to winter (26.6% [1138/4279]) and spring (27.9% [1381/4943]) (*p* < 0.001). Considering 21-year studied period, RVA mean monthly detection rates ranged from 14.2% in January (summer) to 30% in September (spring) (Fig. 1b).

### Rotaviruses A genotype distribution in Brazil before and after vaccination with Rotarix®

**Fig. 3:**
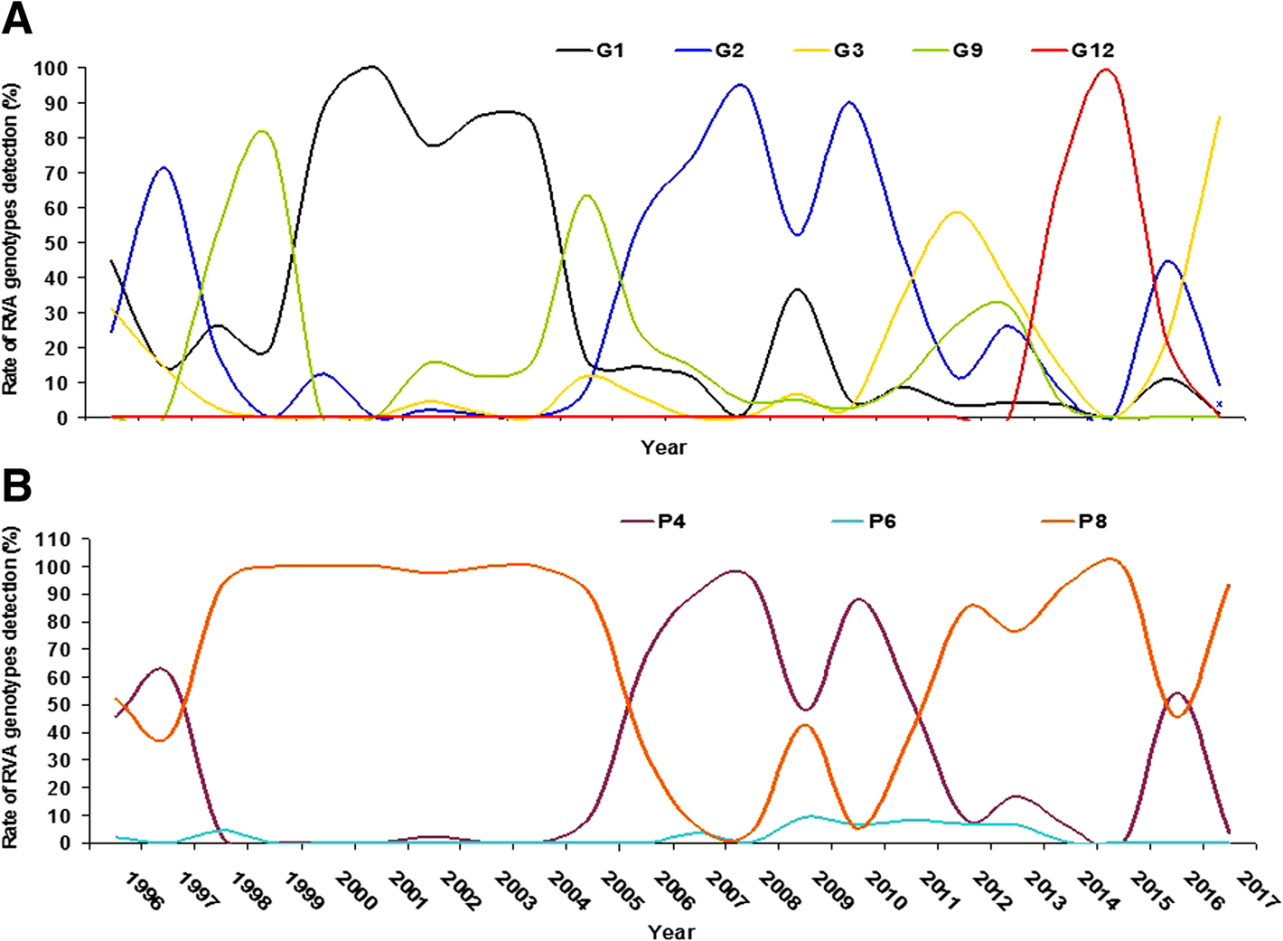
**a** and **b** Laboratory-based rotaviruses A (RVA) surveillance in Brazil, 1996–2016, in 22 out of 27 Brazilian states. Genotypes G (3A) and P (3B) rate distribution

From 3555 RVA-positive samples identified from 1996 to 2016, 2580 (72.5%) were successfully genotyped, being 855 from the pre and 1725 from the post-vaccination period. As presented in Table 2, the detection frequencies of major RVA genotypes in the pre and post-vaccination periods, respectively, was as follows: G1P[8]/G1P[Not Typed], 48.8% (417/855) and 6.4% (118/1835) (p < 0.001); G2P[4]/G2P[NT], 5.5% (47/855) and 45.7% (838/1835) (p < 0.001); G3P[8]/G3P[NT], 6.4% (55/855) and 13.8% (253/1835) (p < 0.001); G9P[8]/G9P[NT], 27.8% (238/855) and 8.3% (152/1835) (p < 0.001); G12P[8]/G12P[NT], 0% (0/871) and 13.6% (249/1835) (p < 0.001). The frequency of atypical G/P combinations, mixed infections or not G-typed strains was 10.9% (93/855) in the pre-vaccination period and 11.8% (217/1835) in the post-vaccination period (*p* = 0.516). RVA genotypes fluctuated in a cyclic manner, with sharp peaks of G2P[4] detection along variable intervals. These peaks were interspersed by the predominance of P[8] circulation, with an interchange of G-genotypes between G1 and G9 in the earlier years, and more recently between G12 and G3 genotypes. The second G2P[4] peak of detection was observed starting months before RV1 introduction, and lasted for the following five years (Fig. 3).

### Rotaviruses A infection and Rotarix® vaccination status

We selected children below the age of 48 months with known vaccine status to assess the association between RVA positivity and RV1 vaccination. Vaccination data became part of the epidemiological records as of March 2006. Considering only children eligible for vaccination (i.e. those who were two months old as of March 2006) 4384 had a known RV1 vaccination status and were aged 4–48 months. From this group, 3090 were fully vaccinated with two doses, 544 were partially vaccinated with one dose and 750 were not vaccinated; RVA positivity in these groups was 547/3090 (17.7%), 123/544 (22.6%) and 222/740 (30%), respectively. Table 3 presents detection rates of RVA by age groups in distinct vaccination status. RVA positivity was significantly lower among vaccinated infants aged 4–11 months (OR = 0.41; 95% CI = 0.29–0.59; *p* < 0.001), among children aged 12–24 months (OR = 0.52; 95% CI = 0.39–0.71; *p* < 0.001) and among patients aged 25–48 months (OR = 0.68; 95% CI = 0.49–0.93; *p* = 0.017). Comparing the positivity rates between unvaccinated and those vaccinated with only one dose, no statistically significant differences were observed. Among RVA-positive children aged 4 to 48 months and with known vaccine status, in 733 it was possible to determine the RVA genotype. Comparing children vaccinated with two doses with unvaccinated children, it was observed that the detection rate of G12P8/P[NT] was higher in the group of vaccinees, while G2P[4]/P[NT] was more frequent among those not vaccinated (Table 4).

**Table 3:** Detection rates of rotavirus A in children with diarrheal disease by age group and Rotarix vaccination status in Brazil, 2006–2017, through laboratory-based surveillance

	RVA^a^ positivity rate	Odds ratio	95% CI^b^	*p*-value
Age group / Vaccination status				
*4–11 months*				
Not vaccinated	58/237 (24.5%)	1		
One dose	63/323 (19.5%)	0.74	0.50–1.12	0.176
Two doses	125/1052 (11.9%)	0.41	0.29–0.59	< 0.001
*12–24 months*				
Not vaccinated	77/260 (29.6%)	1		
One dose	34/156 (21.8%)	0.66	0.41–1.05	0.086
Two doses	253/1395 (18.1%)	0.52	0.39–0.71	< 0.001
*25–48 months*				
Not vaccinated	87/253 (34.4%)	1		
One dose	26/65 (40%)	1.27	0.72–2.22	0.399
Two doses	169/643 (26.3%)	0.68	0.49–0.93	0.017

^a^Rotavirus A; ^b^Confidence interval

**Table 4:** Detection rates of distinct rotavirus A (RVA) genotypes among RVA positive children aged 4–48 months with known Rotarix vaccination status, Brazil, 2006–2017

	RV1^a^vaccination status			
	Two doses (*n* = 434)	One dose (*n* = 103)	Not vaccinated (*n* = 196)	*P*-value^b^
RVA^c^ Genotype				
G1P [8]/G1P[NT^d^]	34 (7.8%)	7 (6.8%)	11 (5.6%)	0.403
G2P [4]/G2P[NT]	222 (51.2%)	53 (51.5%)	125 (63.8%)	< 0.001
G3P [8]/G3P[NT]	62 (14.3%)	19 (18.4%)	29 (14.8%)	0.902
G9P [8]/G9P[NT]	31 (7.1%)	10 (9.7%)	13 (6.6%)	0.867
G12P [8]/G12P[NT]	85 (19.6%)	14 (13.6%)	18 (9.2%)	0.001

^a^Rotarix, ^b^Two doses vs. not vaccinated; ^c^Rotavirus A, ^d^Not typed

## Discussion

The current study demonstrates, by laboratory-based surveillance, a decrease in the frequency of infection with RVA in children presenting with DD after RV1 implementation in Brazil. As recently reviewed, some studies have demonstrated the impact of universal anti-RVA vaccination in Brazil; significant declines of diarrhea-associated hospitalization rates among children ≤5 years-old and infants have been described [23–28]. The present study demonstrated that the reduction in the frequency of RVA infection occurred mainly among children aged 4–11 months-old and 12–24 months-old. The main goal of RV1 vaccination is to prevent severe RVA infections during the first two years of life, and it is well known that DD is more severe in age groups aged less than 24 months-old, the group in which hospitalization occurs due to severe dehydration leading to more frequent deaths. Therefore, it was expected that the main impact of vaccine introduction was likely to occur in age groups less than two-year old. Several studies have shown that RV1 induced immunity protects children from RVA infection in the first two years of life [21, 23, 24, 29].

Interestingly, after RV1 introduction, RVA-positivity showed an increasing trend in children aged 25–48 months-old. Our data are consistent with data reported in the USA, that also demonstrated a shift in the age group distribution of RVA infections, following the introduction of the anti-RVA vaccination [30]. The changes in the age at which children are more likely to become infected with RVA should be considered a beneficial effect of the vaccine.

A somewhat cyclical pattern of genotype circulation was observed, with a 10-year interval between two G2P[4] detection peaks. We observed a long cycle, where DS-1 like and Wa-like genotypes alternated in a 10-year interval and short cycles, where Wa-like genotypes, including G1, G9, G3 and G12 alternated at 2–3 year intervals. The peak of genotype G9 observed in 2005 was mostly attributed to a large outbreak of DD that affected more than 12,000 patients in the state of Acre, Amazonian region of Brazil [31]. At that time, the epidemic of DD was associated with RVA, mainly with genotype G9P[8]. In the same year, other studies described the high circulation of the genotype G9 worldwide. Important changes in RVA genotype distribution have been reported in many countries in the last decades. Among these changes, it is worth pointing out the emergence of G9P[8] in the late 1990s, becoming a very frequent genotype together with G1P[8]. Nonetheless, the most striking global shift in RVA genotype distribution was the reemergence of genotype G2P[4] twelve years ago, shortly preceding and just after implementation of RV1 introduction in Brazil, as well as in countries which did not implement universal RVA vaccination. The fact that RV1 efficacy and effectiveness against genotype G2P[4] is somewhat lower than that observed for Wa-like strains has led to the hypothesis that the long period of G2P[4] predominance could be related to vaccination with RV1 [18–21].

The putative influence of vaccination on the temporal cycling of RVA genotypes was analyzed, and demonstrated an alternation between P[8] and G2P[4]; in turn, G1P[8] and G9P[8] also alternated with each other [32]. However, it should be observed that distinct genetic variants of G2P[4] circulated between 2005 and 2011 in Brazil, and no evidence of selective pressure imposed by the RV1 massive vaccination was observed [33, 34]. In addition, the comparison of G2[4]/G[NT] detection rates in vaccinated and unvaccinated children does not suggest that breakthrough infections have occurred more frequently by this genotype. Interestingly, this was observed with G12P[8]/P[NT], which was detected more often among vaccinates than among non-vaccinates, suggesting some level of vaccine escape for this genotype. RV1 vaccine coverage in Brazil increased from 87 to 95% between 2011 and 2015, having decreased to 88 and 77% in 2016 and 2017, respectively.

The second period of G2P[4] predominance in Brazil lasted 5 years. A recent study performed in Brazil, revealed that new variants of G2P[4] started circulating in Southeastern, Northeastern and Southern regions in 2008, and Northeastern and Southeastern regions in 2010 [34]. It was observed that the re-emergence of G2P[4] was a global phenomenon, and was reported even in countries that had not introduced anti-RVA vaccine [35, 36]. It should be pointed out that in Argentina, a neighbor country where universal vaccination against RVA was implemented in 2015, long lasting predominance of G2P[4] strains started in 2004, and extended until 2011 [37, 38].

Although the re-emergence of G2P[4] appeared not to be associated with the onset of heterotypic vaccination with RV1, we cannot exclude that the massive predominance of G2P[4] in Brazil from 2006 to 2010 may have been influenced by vaccination with RV1. Nevertheless, G2P[4] could not stay for more than 5 years in the environment of vaccinated children, possibly due to the natural induction of homotypic immunity and depletion of the susceptible population. Another noteworthy finding of our study is the re-emergence of G3 from 2011 onwards, replacing G2 predominance after its exhaustion. The G3P[8] genotype has been detected in a higher frequency in the USA, Australia and other countries in the years that followed massive vaccination with RV5 [39]. Reemergence of G3P[6] and G3P[8] was also reported between 2011 and 2012 in Northern Brazil [40]. In the last year of our observation period (2017), another significant increase in the detection rate of G3P[8] was observed. Also observed was a peak of G12P[8] in 2014 and 2015. Luchs et al. (2016) reported a countrywide spread of genotype G12P[8] in the years of 2014 and 2015 in Brazil [41]. Moreover, a global G12 emergence has been observed in the last five years [42].

Our data demonstrates that after RV1 introduction, RVA has been detected in significantly higher frequencies among non-vaccinated children compared to vaccinated ones. These differences were greater in children aged 4 to 11 months, followed by children aged 12 to 24 months. Even in children older than 24 months the RVA detection rates was significantly lower in vaccinated than in non-vaccinated children.

Our study design has limitations, since the health services spontaneously send fecal samples, and consequently there was no systematic sampling in space and time, making data susceptible to bias. However, the results, because they are comprehensive and have been generated by the official surveillance system, shed light in the RV1 vaccination impact in Brazil, and its putative influence in the burden of RVA in the country.

Monitoring other DD viral agents, especially norovirus – detected in high frequency in children with DD – is a current challenge in this new scenario. Continuous viral surveillance must be carried out in Brazil to monitor the circulation of distinct RVA genotypes and other enteric viruses.

## Conclusions

Using data from laboratory-based surveillance, we described RVA molecular epidemiology in Brazil, after a decade of RV1 implementation in Brazil’s NIP. RVA infections are substantially less frequent in children aged less than two years, the most susceptible age group to develop DD complications, such as hospitalization and death. During the studied period (1996–2017), RVA genotypes circulation have alternated with significant decrease of P[8] detection in the post-vaccine period. It was also observed a peak of G12P[8] during 2014 and 2015, and two peaks of G3P[8] detection in 2012 and 2017.

## Abbreviations

DD: Diarrheal diseases
HAstV: Human astroviruses
NIP: National immunization program
NoV: Noroviruses
OR: Odds ratio
PAGE: Polyacrylamide gel electrophoresis
RRRL-LVCA: Regional Rotavirus Reference Laboratory - Laboratory of Comparative and Environmental Virology
RT-PCR: Reverse transcription-polymerase chain reaction
RV1: Monovalent rotavirus vaccine
RVA: Rotaviruses A
